# The role of male quality in sequential mate choice: pregnancy replacement in small mammals?

**DOI:** 10.1098/rsos.240189

**Published:** 2024-07-03

**Authors:** Lea Vodjerek, Filippa Erixon, Clara Mendes Ferreira, Jörns Fickel, Jana A. Eccard

**Affiliations:** ^1^ Animal Ecology, Institute for Biochemistry and Biology, University of Potsdam, Potsdam, Germany; ^2^ Molecular Ecology and Evolution, Institute for Biochemistry and Biology, University of Potsdam, Potsdam, Germany; ^3^ Department of Evolutionary Genetics, Leibniz Institute for Zoo and Wildlife Research, Berlin, Germany; ^4^ Berlin-Brandenburg Institute of Advanced Biodiversity Research (BBIB), Berlin, Germany

**Keywords:** sexual selection, sequential mate choice, male quality, pregnancy replacement, pregnancy termination

## Abstract

Females mainly increase their reproductive success by improving the quality of their mates and need to be discriminative in their mate choices. Here, we investigate whether female mammals can trade up sire quality in sequential mate choice during already progressed pregnancies. A male-induced pregnancy termination (functional ‘Bruce effect’) could thus have an adaptive function in mate choice as a functional part of a pregnancy replacement. We used bank voles (*Myodes glareolus*) as a model system and exchanged the breeding male in the early second trimester of a potential pregnancy. Male quality was determined using urine marking values. Females were offered a sequence of either high- then low-quality male (HL) or a low- then high-quality male (LH). The majority of females bred with high-quality males independent of their position in the sequence, which may indicate a pregnancy replacement in LH but not in HL. The body size of the second male, which could have been related to the coercion of females by males into remating, did not explain late pregnancies. Thus, pregnancy replacement, often discussed as a counterstrategy to infanticide, may constitute adaptive mate choice in female mammals, and female choice may induce pregnancy replacement in mammals.

## 1. Introduction

Sexual selection theory indicates that in most animal species there is a disparity in parental investment where males invest less in their offspring compared with females [1]. Choosing an attractive male can be beneficial for female’s fitness, considering that the genes that a male possesses could increase offspring viability and/or mating success [2]. Most genetic models of mate choice focus on heritable aspects of mating success and viability, but there are also direct benefits that would favour mate choice [3–6], such as fertilization ability or fecundity [3,5], food provisioning [7], parental abilities [1,8] or protection against harassment [1,4,9]. Females may further gain indirect benefits by choosing males from which their offspring are likely to inherit ‘good genes’ that would improve female’s fitness [2,10–14]. Males, in turn, develop traits and behaviours that demonstrate their ability to provide both direct and indirect benefits, and females evolve preferences for these traits [15].

The prevailing belief is that the winners in male-to-male competition are of higher quality and that it would be beneficial for females to mate with them [2,16]. As a result, male–male dominance, and traits that demonstrate it, such as large body size, strong signals of fighting powers, as well as territorial and marking behaviours are believed to play a crucial role in female mate choice. Female preferences for dominant males have been documented in many species [17–20], and in some cases, females may even provoke competition among males and then mate with the winner [16]. Females encounter males simultaneously or sequentially, hence females’ decision to mate or remate if males are encountered sequentially, will have an impact on their fitness [21]. Females may relate a mate to sexually selected characteristics of the male they previously encountered or mated with [22–25]. The trade-up hypothesis has thus been proposed as a female strategy when males are encountered in sequential order [5,26–28], predicting remating only if the second male is of superior quality compared with the first.

Meanwhile, in order to increase their reproductive success, males can coerce females into mating with them in the form of forced copulation, harassment or intimidation [29]. Consequently, there may be a strong selection for male traits such as increased body size that would intensify males’ ability to force females into copulation [30]. With no other females around (as in an isolated pair situation), the frequency of harassment by males, and also the intensity of male mate-guarding [30], is potentially higher than when other females are around [31].

Interruption of early pregnancy (before implantation), also known as pregnancy block or ‘Bruce effect’ [32–34], has been discussed to reduce a female’s investment into offspring that might afterwards be killed by an unknown, invading male [35–37]. Pregnancy block has been recorded under both laboratory and wild conditions in carnivores [38], primates [39,40], rodents [32,34,41,42] and ungulates [43]. While female mice block pregnancy before implantation [32], other mammalian species, for instance, voles and rats, can obstruct pregnancies at later stages [42,44–48]. Male-induced pregnancy termination was initially viewed as a form of post-copulatory male–male competition [1,49,50], but here we propose that it might function as a mechanism of post-copulatory (and in some species post-implantation) mate choice for females, as previously suggested [35]. Hitherto, research has mainly focused on the different proximate, physiological mechanisms [51] or ultimate reasons relating to sexual conflict (for review, see [52,53]). This focus on the proximate mechanism may have somehow obstructed the ultimate consequences of pregnancy termination: to make way for a new pregnancy with potentially superior offspring. In laboratory mice, socially dominant males induced higher rates of pregnancy blocks than socially subordinate males [54]. However, the ultimate proof that females can improve the quality of their offspring by sequential mate choice has been missing.

To investigate the mate choice component of pregnancy turnover, we studied mate choice in small mammals. We presented two males sequentially to a female, whereby we exchanged the breeding male at a progressed state of a potential pregnancy, here in the early second trimester. We hypothesized that: (1) if females could trade up sire quality by pregnancy replacement, they exchange pregnancy at the cost of delaying offspring births. Thus, given the sequential choice between males of different qualities, we would expect a female to deliver offspring later in a treatment where the second male was of superior quality relative to the first, compared with a treatment where the second male was of inferior quality compared with the first. Alternatively, if the pregnancy replacement was not due to the female choice among the two breeding males, we would expect delayed deliveries in relation to the properties of the second male: (2a) its absolute quality (regardless of the first male’s quality) or (2b) its body size relative to the female’s body size (indicating strength to coerce the female into mating). To test these alternatives, we added a treatment in which both males were of the same high quality and measured body size dimorphism between females and second males, assuming that only relatively larger males could coerce females into copulations. To ensure the reproducibility of the effects [55–57] of male quality on sireship, we combined two independently collected datasets. There is a growing awareness that the rigorous standardization of experimental conditions may contribute to poor reproducibility of results in animal studies. Instead, systematic heterogenization of the experimental environment has been proposed as a tool to enhance reproducibility [55–57]. We included tests on reproducibility in our statistical analysis.

## 2. Methods

Bank voles (*Myodes glareolus*) are small, cricetid rodents, commonly found in forests and scrub habitats throughout Eurasia [58,59]. Females show a preference for high-quality males in simultaneous choice trials [18] and would mate with both males if they are offered sequentially within oestrus [27]. Coercion by males has not been confirmed in matings of captive bank voles [27]. Infanticide has been reported in both laboratory and field populations of bank voles [60], both committed by non-parental males [61,62] and females [63].

For our experiments, we used wild-captured or laboratory-born F1 descendants of wild-captured bank voles. Cages were equipped with enrichment items like egg cartons, toilet paper rolls and a running wheel. The temperature in the holding rooms was maintained between 20 and 22°C and a 16 h : 8 h light/dark period was maintained. Food and water were provided ad libitum. All males and females were reproductively active, females were not pregnant and both males and females were weighed before the experiment. To avoid that males and females were related, we paired individuals that were captured from locations at least 60 m apart (wild captures) or we paired wild-captured and laboratory-born individuals.

### 2.1. Male quality assessment

Male quality was assessed in paired trials using urine marking (UM) behaviour, which is used to classify dominance relationships in rodents [64], and specifically in bank voles [18,65]. As evidenced by the literature, low-quality (LQ/L) males only leave a few concentrated urine spots, while high-quality (HQ/H) males extensively mark large areas with thin, squiggly shaped traces [18,66], which are used to quantify the urine marking value (UMV). Males, however, were not able to interact, but could only use olfactory, and partially visual cues, to detect each other. This particular method has been proven reliable, and a good replacement for the earlier method where males would be allowed to interact, which could lead to some serious injuries [18].

UM behaviour is independent of the opponents’ marking behaviour and identity, and it is consistent over repeated testing [18,27,61]. We, therefore, treat UM behaviour as an indicator of intrinsic male quality rather than a rank relative to the opponent. In simultaneous mate choice experiments, males with higher UMV were chosen over those with lower UMV in females’ first choices [67] and by females in post-partum oestrus [18,27] indicating that UMV is used by females as a proxy for male quality. Further, dominance-related traits such as the weight of preputial glands and UM behaviour were shown to be heritable [68] in bank voles.

### 2.2. Datasets

We generated two datasets in two independent laboratories using very similar methods and animal age groups [57]. However, due to local differences, experimental settings were not identical but differed somewhat in the assessment of male quality and the size of cages used (see differences in table 1).

**Table 1.** Details of datasets: in both studies in bank vole colonies females were subsequently paired with males (first and second) for one week each. In the datasets, females were offered a sequence of either: high- to low-quality male (HL), high- to high-quality male (HH) and low- to high-quality male (LH), in the table referred to as ‘treatments’. The response variable was the timing of births after the removal of the second male (NA, not measured). Each treatment is represented by the number of successful pairings (i.e. the number of obtained litters) within a dataset.

	dataset 1	dataset 2	comparison (test, significance)
place	Potsdam, Germany	Konnevesi, Finland	
years	2022	1999, 2001	
sample size (F)	13	19	
sample size (M)	18	32	
holding cage size	3250 cm^2^ (Type IV)	1120 cm^2^ (Type III)	
no. males involved HQ/LQ	9/9	23/9	*χ*^2^ = 1.5, *p* = 0.215
male quality assessment	absolute (each male repeatedly)	relative (M1 versus M2)	
weight female (*n*)	20.2 ± 4.6 (*n* = 17)	15.6 ± 1.2 (*n* = 6)	*t* = −4.12, *p* = 0.003
weight male (*n*)	24.8 ± 3.4 (*n* = 18)	23.4 ± 4.1 (*n* = 14)	*t* = −1.05, *p* = 0.305
**hypotheses and treatments**			
1) mate choice: HL–LH	13 HL–8 LH	4 HL–8 LH	*χ*^2^ = 1.48, *p* = 0.223
2a) second male quality: HH–HL	0 HH–13 HL	7 HH–4 HL	
2b) weight difference: second male–female	21 MF pairs	5 MF pairs	

The first dataset was obtained in 2022 in Germany. Quality scores were obtained before assigning males to different treatments and pairing them with females. Each male was tested three times against random male opponents in short rounds (2 h). Tests were conducted in two connected arenas (L × W × H = 42 × 30 × 20 cm) divided by a 42 cm long plastic wall with 5 mm holes. A repeatable UM behaviour (traces or puddles) was used to classify them as HQ or LQ (repeatability category: *R* = 0.791, s.e. = 0.194, CI = 0.274, 0.993, *p* = 0.001). HQ males (*n* = 9) marked 56% ± 24% of the arena floor (means of three tests over 2 h each), LQ (*n* = 9) marked 24 ± 19%. Further, 8 of the 18 males were used twice in the experiment, in combination with another male and another female.

The second dataset was collected in 1999–2000 in Finland. UMV of bank vole males were obtained *a posteriori* after the successful reproduction of the female. Here, we also found pairings of two males that were both categorized as HQ, thereby allowing the inclusion of a third treatment (HH, Hypothesis 2a). UMV of males were obtained as a relative measure, related to rank order and obtained from exactly the two males offered to the female [18,69]. UMVs were assessed by keeping the two candidate males overnight in a 60 × 40 × 34 cm (L × W × H) arena divided into two sections by wire mesh with a 60 cm border. Males categorized as high quality (*n* = 23) marked 79% ± 13% (mean ± s.d.) of the arena floor overnight, and males categorized as low quality (*n* = 9) marked 46% ± 11.3%. The proportion of area marked differed from the first dataset, since (i) males were kept in the marking arena for longer, and (ii) the size of the arenas differed with a longer border in the arenas. With a difference of greater than 10% of the absolute area marked, males were considered of different quality. Twelve of the tested male pairings qualified as different quality (HQ–LQ (HL) or LQ–HQ (HL)), and in seven pairings both males were high quality (HQ–HQ (HH)). Six individual males appeared twice in this dataset. Knowing that UMVs are repeatable and independent of the opponent [61,70,71], we assume that a ranking approach would categorize the same male as HQ (or LQ, respectively) as an intrinsic-quality approach.

### 2.3. Mate choice testing procedure

Each female was paired with the first male for a duration of 7 days (5–8 days in dataset 2), after which the first male was removed and replaced with the second male, which stayed with the female for another 7 days. For the experiment, males were placed into females’ cages to mimic the natural situation where males would enter females’ territory. On an experimental day 14, pairs were separated, and all animals were transferred back into their single-home cages. The exchange of males at the end of the first trimester was assumed to allow females to either continue the pregnancy with the first male or undergo a pregnancy replacement and breed with the second male. We also considered an alternative explanation: that females were not breeding with the first male at all, but only bred with the second male. However, in caged bank vole pairs the proportion of females breeding did not increase with additional pairings [72], so we assume that females breed with every male (if they can), and in laboratory mice, the breeding rates did not differ between pairings with HQ or LQ males [54].

**Figure 1.**
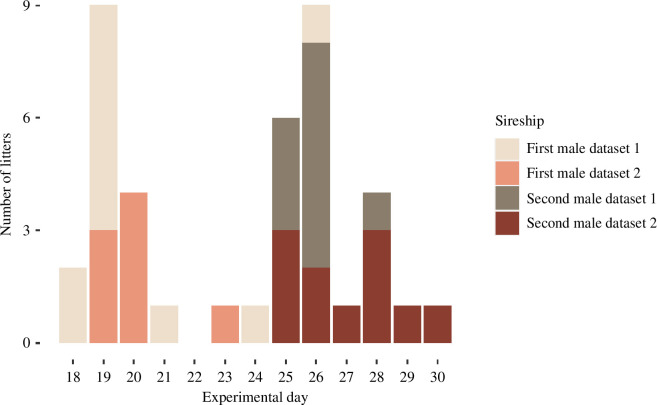
Birthday, total number and distribution of bank vole litters across experimental days 18–30 with sequential mate choice. Females were introduced to the first male at day 0. The first male was removed after 5–8 days (mean 7 ± 1 day) and replaced by the second male. Litters were assigned to males based on birth date, more than18 days after the introduction of the respective male (bank vole pregnancy lasting 20 ± 2 days [73], dataset 2), and by genetic paternity assignment during the critical period (days 25–29, dataset 1). Births are colour coded by assignment and dataset.

Cages were controlled daily for births between experimental days 18 and 32. Bank vole pregnancies last 20 ± 2 days (mean ± s.d. [73]), bank voles have a cycling oestrus every 3–4 days [72], and most births (82%) occur after 18–22 days after caging voles in pairs, even if partners are not separated later [72], i.e. births follow a right-skewed distribution. Births in our experiment occurred predictably in a bimodal distribution, with two right-skewed peaks at 19 and 26 experimental days (figure 1). Thus, litters being born earlier than 18 days after the male turnover could be unequivocally assigned to the first male, while litters 18 days after male turnover (more than 24 experimental days) were very likely sired by the second male.

### 2.4. Genetic paternity analysis

In dataset 1, we challenged our paternity assignment for the litters born in the second peak (days 25–29, figure 1), using genetic paternity analyses. Further, we only tested genetic samples from dataset 1 as samples for the animals from dataset 2 were not obtained when the experiment was conducted. Tissue samples were collected from parents and litters by a small ear biopsy. DNA extraction and microsatellite amplification were done according to the protocol of Gockel *et al.* [74] for up to seven microsatellite loci MSCg—4, MSCg—7, MSCg—9, MSCg—15, MSCg—18, MSCg—24 and MAR—113 [74,75].

Parentage was assigned with the software COLONY v. 2.0.7.0 [76], within each family separately. The analyses were run assuming a polygamous mating system with known mother–litter relations, with each analysis being replicated 20 times within the software and independently run six times using different seed numbers, with all runs showing consensus. Since all parents were sampled, we used a 1.0 proportion of sampled parents. The inclusion of allelic frequencies or possible rate of null alleles, as calculated in the packages ‘adegenet’ [77] and ‘PopGenReport’ [78], did not change the paternity assignment. In cases, where COLONY could not estimate paternity (e.g. potential genotyping errors), we further calculated the proportion of shared alleles and the pairwise relatedness of father–litter relations using the R packages ‘adegenet’ and ‘related’ [79]. If the proportion of shared alleles was higher than 0.35 and pairwise relatedness higher than 0.25 or overall higher in one of the putative fathers, we would consider that male as the father.

We assigned paternity to 10 out of 11 (one litter could not be analysed) families using genetic parentage analyses and confirmed 9 out of 10 of our assignments (90%). One litter had a change in the assignment. In the litter that could not be analysed, we assigned paternity based on the birthday, as this method has been proven reliable, as additionally indicated by the genetic paternity analyses. Multiple males sired none of the litters. One family did not have any of the males confirmed as fathers, due to missing genetic samples. In one family, only one of the offspring had its paternity identified with high confidence (confirmed in COLONY). As we did not find other confirmation of multiple paternity in any of our families, we assumed this male to have fathered the entire litter.

### 2.5. Statistical analysis

We compared the proportion of late litters (i.e. sired by the second male; binomial) between the treatments, using generalized linear models [80], with treatment and the weight difference between the female and the second male as explanatory variables for the respective hypotheses. For the alternative hypotheses, we always included treatment in the analysis.

To investigate the reproducibility of the potential effects of male quality on sireship among datasets, we investigated the differences among datasets and the interaction of datasets with treatment. If the strength, direction or presence of an effect would depend on the testing environment, this could be picked up by a statistical interaction term [55–57], which we investigated in a separate model due to restricted sample size. Female ID was initially added as a random factor, since eight females were used twice in the experimental pairing. Based on preliminary results from the generalized mixed effect model the random factor (female ID) did not contribute to the variance explained and did not improve the model fit (model comparison: delta AIC = 2) [81]. Therefore, we excluded the random factor and changed from generalized linear mixed model (GLMM) to generalized linear model (GLM) [81]. From the models, we report log-likelihood and effect sizes, as well as the variance explained.

Associations between the quality and weight of the males were investigated in both datasets, using *χ*^2^ tests and *t*-tests. Statistical analyses were performed with R v. 4.2.2. using R-studio (RStudio Team 2022), and the packages ‘lme4’ [82], ‘car’ [83] and ‘rptR’ [84].

## 3. Results

**Figure 2.**
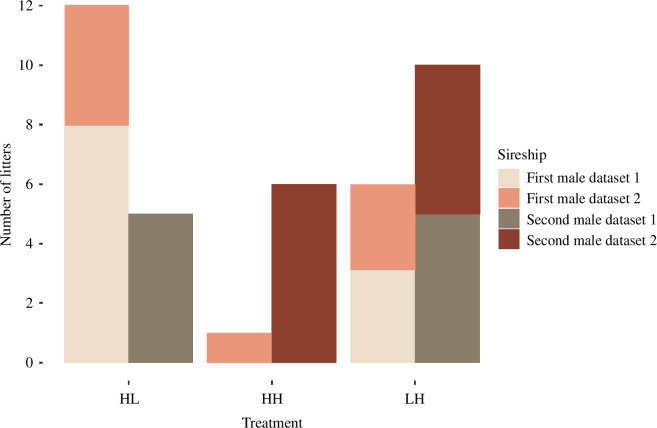
The number of bank vole litters sired by the first and second males in a sequential mating treatment, where each male stayed with the female for one week. Males differed in quality (HL = high–low quality; HH = high–high quality; LH = low–high quality), and delivery of litters sired by the second male probably included a pregnancy replacement, indicating mate choice. Births are colour coded by assignment and dataset.

In the 33 mate choices among males of different quality (both LH and HL treatments), 67% of females (overall) reproduced with the HQ male (figure 2). No interaction between treatment and datasets was detected (*χ*^2^ = 2.37, d.f. = 1, *p* = 0.124), hence the effect of male quality on late litters was reproducible among the two datasets. Therefore, we removed the interaction term from the analyses and used a simpler, better-fitting model. The proportion of late litters (i.e. sired by the second male; overall: 53%) differed significantly between the three treatments (GLM, *n* = 40, *χ*^2^ = 8.33, d.f. = 2, *p* = 0.01, table 2, figure 2).

**Table 2.** The proportion of late litters between treatments (Hypotheses 1 and 2a) and as a response variable to the weight difference between the second male and the female as an indication of coercion (Hypothesis 2b). Shown are the results for the sequential mate choice experiment with the proportion of late litters as the response variable. The number of individuals is stated. In the model, we have compared different treatments against each other (Treatment HL–LH: HQ–LQ versus LQ–HQ; with the first-factor level as a reference). *R*^2^ values are based on fixed factors. Statistically significant *p*-values are shown in bold.

dataset	response variable	fixed effects	estimate	s.e.	*Z*	*p*
1+2 (*n* = 40), *R*^2^ = 0.26	late litters	intercept	−1.5029	0.885	−1.669	0.089
		treatment HL–HH	3.293	1.396	2.36	**0.01**
		treatment HH–LH	−1.656	1.261	−1.313	0.189
		treatment HL–LH	1.639	0.818	2.005	**0.04**
		dataset (2 versus 1)	0.789	0.849	0.929	0.353
1+2 (*n* = 26), *R*^2^ = 0.93	late litters	intercept	−0.725	0.666	−1.089	0.276
		weight difference	0.08	0.095	0.826	0.41
		treatment HL–LH	0.895	0.963	0.929	0.353

If the HQ male was offered to a female after an LQ male, a higher proportion of females reproduced with the second male (63% of late litters) compared with when the LQ male was offered after the HQ male (29% of late litters; *post hoc* Tukey test: *χ*^2^ = 3.71, *p* = 0.05). Further, if both males were of HQ, the proportion of late litters was higher (86%) compared with when the LQ male was offered after the HQ male (*χ*^2^ = 6.77, *p* = 0.009). The (high) proportion of late litters stayed high independent of the quality of the first males, as long as an HQ male was second in pairing order (*χ*^2^ = 1.36, *p* = 0.244, comparing HH and LH treatment).

The dimorphism between the second male and the female ranged from −5 g (larger female) to 15 g (larger male, mean weight difference 4.6 g ± 5.01 g) body weight difference but did not explain the proportion of late births (GLM, *n* = 26: *χ*^2^ = 0.71, d.f. = 1, *p* = 0.399, table 2). HQ males were not heavier (24.6 ± 3.9) than LQ males (23.6 ± 3.3; *n* = 32, *t* = 0.74, d.f. = 26.7, *p* = 0.463).

## 4. Discussion

Based on a higher proportion of litters (67%) sired by HQ males in treatments with different male qualities (HL and LH) we were able to demonstrate that females preferred HQ males over LQ ones (Hypothesis 1). The effect of male quality on sireship in subsequent matings was reproducible across two different datasets [55–57] that were collected in different years with slight differences in breeding conditions. In contrast to earlier studies, in our experimental setting females could not choose between two males presented simultaneously but only between two males presented sequentially, and without prior knowledge if the second choice would be offered to the female. If the female would actively choose to reproduce with the second male, this would only be possible through a pregnancy replacement, and we suggest that the ‘Bruce effect’ is the potential mechanism. We suggest that mate choice in a mammal can induce pregnancy replacement.

Our findings would be in line with Halliday’s [5] hypothesis that suggested females might breed with the first male they encounter, just to ensure fertilization, but if the second male becomes available, and is better than the previous, females may choose to remate in order to trade up [5]. Different from earlier findings in allied rock wallabies (*Petrogale assimilis*), who trade up for low-quality social partners by searching for extra-pair copulations [85], crickets [23], sticklebacks [86] and newts [87], which show sequential mate choice within one mating bout, our data indicates sireship of the better male even if at the cost of giving birth to the offspring at a later time and losing the initial investment into early pregnancy. Meanwhile, it is also possible that females were not choosy, but males of higher quality have a greater ability to manipulate females to terminate the pregnancy and remate, in comparison with low-quality males. We did not find evidence that size differences between males and females could affect the sireship, and no differences were found in the mating behaviour of dominant and subordinate male bank voles [27]. Whether or not males of different qualities differ in courting behaviour has not been investigated.

With a high number of late litters both in the HH and LH treatment, pregnant females apparently base their remating decision rather on the absolute quality of the second male encountered than on the comparison of the relative quality of the first male versus the second male (Hypothesis 2a), similar to the finding in house mice where the rate of pregnancy blocking was related to the dominance of the second male rather than to that of the first male [54]. A female may not be able to remember the quality of a previous male [88], but this seems unlikely since the first male was replaced with the second within a few hours. Sireship in multiple male matings can be biased towards later mates [27,89], but the results from sequential mate choice within one oestrus can hardly be compared with results in our progressed-pregnancy setting. Thus, our data indicate that the absolute property of the second male contributes to late pregnancies, which can be interpreted as female choice, male manipulation or a mixture of both. The higher paternity success of dominant males found in the free female choice experiment (males were not allowed to leave their compartments) confirms that social status is indeed a signal of male quality in bank voles and therefore should be favoured by sexual selection [27]. Investigating other properties of the second male in our datasets, we found no evidence of coercion (Hypothesis 2b).

Although in bank voles there is no evidence for coercive matings (I Klemme 2005, personal observation) and bank voles are sexually monomorphic, the artificial situation of a caged pair could have produced artefacts [90] in which body size matters. The weight difference between the second male and the female did not explain the occurrence of late pregnancies, hence, there is no evidence that the later males coerced females into mating (Hypothesis 2b). A conflict of interest between the sexes is likely to occur if males can coerce females into mating [1,91]. Females may gain an increase in their fitness by delaying or refusing mating if they would be able to mate with another, superior male [30]. Males, however, will gain a fitness increment if they mate with as many females as possible [2], therefore male traits such as increased body size might be favoured by selection. Increased body size enhances a male’s ability to coerce females into mating, and consequently results in higher fitness. In nature, there is a chance that females can segregate spatially from the harassing male [92], but experimental bank vole pairs were kept in a cage, and there was no way to avoid the male. Still, size dimorphism did not explain late pregnancies, we, therefore, do not consider coercion or harassment the defining mechanism.

Bank voles, like many other mammals, are at risk of infanticide. The occurrence of infanticide has been observed in both laboratory and wild populations, committed by non-parental adults of both sexes [60,62,63]. In this experiment, males were removed before the pups were born. However, females might consider future infanticide by the non-parental males and engage in pregnancy replacement to avoid the possible damage to their offspring. If the ‘Bruce effect’ would serve as a counterstrategy to avoid infanticide (for review, see [53,91]), infanticidal tendencies of males would be related to male quality (they are in house mice [54] but not in voles [61]), and infanticidal tendencies would be related to the probability of pregnancy replacement, which has not been investigated so far.

With 71% of litters sired by the first male in the HL treatment, we can safely assume that pregnancy rates of caged pairs were generally high in first matings, and we further assume that most of the delayed litters observed in HH and LH treatments included a pregnancy replacement. Further, there is no evidence that pregnancy rates were lower if the first male was of lower quality than if it was of higher quality [54]. It was shown that an early-stage pregnancy is not very costly for bank voles [93], but the temporal costs of delaying reproduction may be high for a short-lived small mammal. A time constraint can alter the choosiness of the female, even if there is no explicit cost to waiting [88]. Nonetheless, females could abort the offspring previously sired by a LQ male for a subsequent conception from a HQ male [37]. Therefore, pregnancy replacement would be adaptive only in the case when the HQ male is encountered after a LQ male and would indicate a post-implantation mate choice for females. As previously proposed, the fitness gain from choice, and hence optimal choosiness, should be affected by these variables: the distribution of mate quality, the cost of searching for mates and the chooser quality [22,94–96], and in our study system, the cost of delaying pregnancies.

## 5. Conclusion

In this study, we demonstrated that pregnancy replacement could be a result of sequential mate choice in mammals since late litters were more likely if the second male was of high quality. In conclusion, we found that the proportion of late births (assumed to come from a second pregnancy) was higher if paired females encountered a high-quality male as a second mate, indicating a pregnancy turnover to achieve a trade-up sire quality in a second pregnancy. Our results did not support the coercion hypothesis for pregnancy replacement. Therefore, with this experiment, we are presenting pregnancy termination as a mechanism of sequential mate choice in a small rodent, which may be an ultimate, adaptive reason for pregnancy replacement in mammals.

## Data Availability

Data available online [97].
